# Human Endogenous Retrovirus Reactivation: Implications for Cancer Immunotherapy

**DOI:** 10.3390/cancers13091999

**Published:** 2021-04-21

**Authors:** Annacarmen Petrizzo, Concetta Ragone, Beatrice Cavalluzzo, Angela Mauriello, Carmen Manolio, Maria Tagliamonte, Luigi Buonaguro

**Affiliations:** Laboratory of Innovative Immunological Models, Istituto Nazionale per lo Studio e la Cura dei Tumori, “Fondazione Pascale”-IRCCS, 80131 Naples, Italy; concetta.ragone@istitutotumori.na.it (C.R.); beatrice.cavalluzzo@istitutotumori.na.it (B.C.); a.mauriello@istitutotumori.na.it (A.M.); carmen.manolio@istitutotumori.na.it (C.M.); m.tagliamonte@istitutotumori.na.it (M.T.)

**Keywords:** human endogenous retroviruses, cancer vaccine, tumor-specific antigens, cancer immunotherapy, hepatocellular carcinoma

## Abstract

**Simple Summary:**

Endogenous viruses are “ancient” viruses that have coevolved with their host species for millions of years, developing strategies to maintain an equilibrium state with their host. In particular, human endogenous retroviruses (HERVs) are permanently integrated and make up over 8% of our genome. Recent studies have shown that the equilibrium between these endogenous retroviruses and our cells can be broken in several conditions, including cancer. HERV reactivation in cancer cells may result in (a) the activation of a viral defense response against cancer, (b) the production of viral proteins that can be recognized as targets by our immune system and (c) the expression of viral transcripts that can be used as therapeutic targets or markers for prognosis. Overall, this may positively impact on cancer immunotherapy strategies.

**Abstract:**

Human endogenous retroviruses (HERVs) derive from ancestral exogenous retroviruses whose genetic material has been integrated in our germline DNA. Several lines of evidence indicate that cancer immunotherapy may benefit from HERV reactivation, which can be induced either by drugs or by cellular changes occurring in tumor cells. Indeed, several studies indicate that HERV proviral DNA can be transcribed either to double-stranded RNA (dsRNA) that is sensed as a “danger signal” by pattern recognition receptors (PRRs), leading to a viral mimicry state, or to mRNA that is translated into proteins that may contribute to the landscape of tumor-specific antigens (TSAs). Alternatively, HERV reactivation is associated with the expression of long noncoding RNAs (lncRNAs). In this review, we will highlight recent findings on HERV reactivation in cancer and its implications for cancer immunotherapy.

## 1. Background

Since January 2021, regulatory agencies, such as the EMA (European Medicines Agency) in Europe and the FDA (Food and Drug Administration) in the U.S. are rushing to approve new tested vaccines for a novel coronavirus strain known as SARS-CoV-2 (severe acute respiratory syndrome coronavirus 2).

SARS-CoV-2 represents a serious threat to public health, as well as to the global economy, due to the impact of the lockdown measures implemented to prevent the spreading of the virus. However, COVID-19 (coronavirus disease 2019) is neither the first nor the last pandemic that we will face in the future [1].

Indeed, viral infections have challenged the human race for millions of years, and paleovirology specifically studies the impact that ancient viruses (i.e., paleoviruses) have had on host species and their genomes [2].

In particular, direct paleovirology studies the host genome in search of “viral fossils”, also known as endogenous viral elements (EVEs), which represent the sign or the remnants of past DNA/RNA virus infections [3].

Interestingly, several lines of evidence indicate that some endogenous viral elements have been co-opted as cellular genes. In particular, virus-derived genes have been discovered in several species where they have gained antiviral function. For instance, the mouse *Fv1* gene, which is derived from a retroviral *gag* gene, provides resistance to the murine leukemia virus (MuLV) [4]. 

Another example of co-option, with non-antiviral purposes, is represented by human syncytins (i.e., syncytin-1 and syncytin-2) in placenta, which derive from the envelope (*env*) gene of human endogenous retroviruses (HERVs), which in turn derive from infections by ancestral exogenous retroviruses [4,5].

Alternatively, indirect paleovirology evaluates the adaptive changes that occurred in host genes in response to ancient viral infections [6]. This is particularly true for genes like TRIM5 (tripartite motif containing 5), the APOBEC (apolipoprotein B mRNA editing enzyme, catalytic polypeptide-like) family and SAMHD1 (SAM domain and HD domain-containing protein 1), endowed with antiviral function, as part of the innate immune system. Interestingly, both direct and indirect paleovirology give us a clue as to how ancient viruses have affected the evolution of their host genomes, as well as their susceptibility to current viruses [7].

The present review will highlight recent findings on human endogenous retroviruses (HERVs), emphasizing their unique aspects of reactivation and expression in cancer, with particular focus on the implications for immunotherapy of cancer.

## 2. Human Endogenous Retroviruses

Human endogenous retroviruses derive from ancestral exogenous retroviruses whose genetic material has been integrated in our germline DNA. HERVs make up over 8% of our genome [8]. 

HERVs are currently classified depending on the amino acid coupled to the tRNA binding the viral primer binding site (PBS) to start reverse transcription. For instance, members of the HERV-K family use a lysine (K) tRNA to prime reverse transcription. Among the known HERV families, HERV-K is the most recently acquired and is further divided into 11 subgroups (i.e., HML-1–HML-11) (Table 1). Nevertheless, further subgroups have been recognized and a revised system for their classification and nomenclature has been recently proposed [9,10,11]. 

HERVs are recognized based on sequence homology with exogenous retroviruses (Table 1). Indeed, like all the other members of the infectious exogenous *Retroviridae* family, HERVs may retain *gag*, *pol* and *env* genes, as well as the two long terminal repeats (LTRs), depending on their evolutionary age. In particular, evolutionarily old HERVs are characterized by extensive accumulation of genetic mutations or gene loss [12].

Several aspects of HERVs’ contribution to chronic diseases, such as cancer, autoimmune and neurological diseases are controversial. In particular, initial evidence proposed a role of etiologic cofactors for HERVs in cancer development through stimulation of cell fusion and immunosuppression by env proteins [13]. However, evidence is accumulating on the protective role of HERVs in certain tumors [14]. These contrasting observations highlight the complex aspects of HERV activation in human diseases, particularly in cancer.

In particular, the study by Lemaitre et al. [15] provides evidence for a role of HERV-K env in promoting transformation and epithelial-to-mesenchymal transition (EMT) in a non-tumorigenic epithelial cell line. The authors found that the HERV-K env cytoplasmic tail was able to activate the ERK1/2 pathway, as well as several transcription factors mostly associated with transformation in melanoma. Conversely, the study by Singh et al. [16] provides evidence for a protective role of HERV-K rec protein. Indeed, the authors found that rec protein may inhibit the EMT process, as well as the invasiveness and metastasis of melanoma. Interestingly, these two seemingly conflicting results might be due to differential expression of two HERV-K alternative splice products, namely, env and rec, and their effect on the EMT process of cancer progression.

Several epigenetic mechanisms may contribute to the regulation of HERV expression in normal tissues and cancer, including DNA methylation, as well as histone modifications [17]. For instance, constitutive DNA hypomethylation is associated with aberrant expression of the ERVWE1/syncytin-1 transcript in seminomas [18]. Similarly, expression of HERV-Fc1 increases in peripheral blood mononuclear cells (PBMCs) upon treatment with trichostatin A (TSA), a histone deacetylase inhibitor (HDACi). On the contrary, TSA does not lead to increased HERV-Fc1 in HEK-293 cells, suggesting a cell-type-dependent effect [17]. Indeed, HERV expression is regulated by complex mechanisms that involve multiple control strategies. In particular, DNA methylation plays a major role in silencing evolutionarily young HERVs, whereas histone methylation represents the major mechanism to silence intermediate-age HERVs [19]. TRIM28 (tripartite motif containing 28) and FAM208A (family with sequence similarity 208 member A), a component of the HUSH (human silencing hub) complex, may contribute to the silencing of young LTR promoters via trimethylation of histone H3 at lysine residue 9 [20,21].

Overall, the evolutionary path towards epigenetic silencing during HERV aging is a multistep process. Indeed, recently integrated LTR elements (i.e., young HERVs), which have high CpG densities, are silenced by DNA methylation at CpG sites. However, spontaneous deamination of methylated cytosines may occur with a consequent C-to-T transition. Therefore, endogenous retroviruses show a progressive loss of CpG sites as a function of their evolutionary age. Consequently, the silencing path switches from DNA methylation to histone methylation (i.e., “epigenetic switch”) in intermediate-age HERVs characterized by low CpG density [19].

Accordingly, different classes of drugs are active in reverting HERV silencing. In particular, inhibition of DNA methyltransferases (DNMTs) by 5-aza-2′-deoxycytidine (5-aza-CdR) may induce the expression of evolutionarily young HERVs. On the contrary, no effects are observed by the single inhibition of G9a, a histone methyltransferase that catalyzes methylation of histone H3 at lysine residues 9 and 27 [22].

Several studies indicate that young LTRs are still repressed by G9a after 5-aza-CdR treatment due to epigenetic switch. Therefore, a combination treatment with 5-aza-CdR plus G9a inhibitor (G9ai) simultaneously removes both repressive mechanisms, resulting in further upregulation of young HERV expression [19,22].

In this scenario, the upregulation of HERVs by dual inhibition of DNA and histone methyltransferases is rapidly becoming of interest for predicting cancer patient response to epigenetic therapies [23,24]. Indeed, cancer immunotherapy may benefit from HERV reactivation induced by epigenetic drugs, and the stimulation of a viral mimicry state (Table 2) [25].

Alternatively, HERV reactivation can be tumor-associated. Indeed, a dysregulated expression of HERVs in human cancers appears to be due to tumor-specific DNA hypomethylation. As a result, HERV reactivation may lead to viral protein synthesis with the generation of newly unexplored tumor-specific antigens (TSAs). These antigens may potently elicit antitumor B and T cell responses, and positively impact on cancer immunotherapy (Table 2) [26].

A third significant aspect of HERV reactivation is associated with the expression of long noncoding RNAs (lncRNAs), as recently observed in hepatocellular carcinoma (Table 2) [27,34].

**Figure 1:**
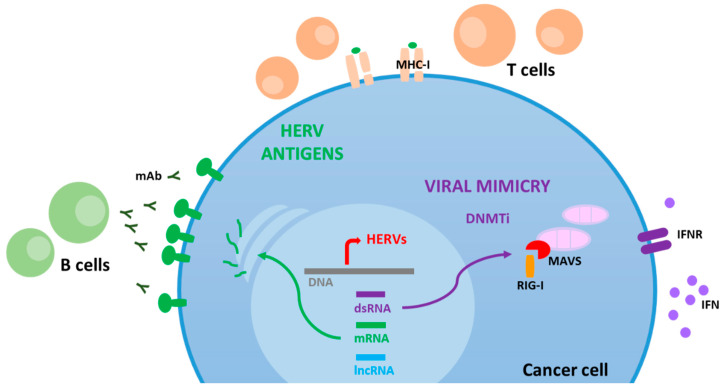
HERV reactivation in cancer cells may result in a viral mimicry state, generation of highly tumor-specific antigens and expression of LTR-activated transcripts, including long noncoding RNAs (lncRNAs).

Overall, HERV reactivation in cancer cells may result in (a) a viral mimicry state, (b) generation of highly tumor-specific antigens and (c) expression of LTR-activated transcripts, including lncRNAs (Figure 1).

## 3. Viral Mimicry State

The overexpression of HERV RNAs (dsRNA) is sensed by intracellular pattern recognition receptors (PRRs) [28,29,35] and may induce a viral mimicry state, leading to an inflammatory environment able to recruit numerous immune cells to the tumor site. Indeed, the inherent DNA hypomethylation in some tumors may result in the activation and overexpression of HERVs. Alternatively, tumors characterized by a low HERV expression can be reverted by epigenetic therapy to overexpress HERV RNA transcripts. Ultimately, the HERV-associated viral mimicry state can provide a synergistic effect with anticancer therapy [36,37].

PRRs represent, indeed, a barrier adopted by the innate immune system against microbial infections. PRRs are able to recognize highly conserved motifs of microbial origin, known as pathogen-associated molecular patterns (PAMPs), as well as damage-associated molecular patterns (DAMPs), initiating a response that culminates with the production of type I interferons (i.e., IFNα and IFNβ) and proinflammatory cytokines [30].

Type I IFN release induces the transcription of several interferon-stimulated genes (ISGs), and the further production of IFN that participate in the activation of the adaptive immune response. In particular, type I IFNs specifically stimulate the expression of MHC class I and II molecules on antigen-presenting cells (APCs) for effective T and B cell activation and differentiation [31].

Two major classes of PRRs, including Toll-like receptors (TLRs) and retinoic acid inducible gene I (RIG-I)-like receptors (RLRs) are known to be activated by endogenous retroviruses in humans and mice [28,29,35,38].

TLRs are expressed on the extracellular membrane (TLR1, TLR2, TLR4, TLR5, TLR6 and TLR11) or associated with the intracellular endosomes (TLR2, TLR3, TLR7, TLR8, TLR9 and TLR10) of macrophages and dendritic cells (DCs). Among them, endosome-related TLRs, including TLR3, TLR7, TLR8 and TLR9, respond to the viral RNA of endogenous retroviruses via the adaptor molecules TRIF (TIR domain containing adaptor inducing IFNβ) and MyD88 (myeloid differentiation primary response protein 88) [32].

In particular, a recent study showed that mice triple-deficient for TLR3, TLR7 and TLR9 presented a strong upregulation of ERV sequences that correlated with virus production and ERV viremia in the absence of an immune activation status [28]. The authors suggest that TLR7 and IRF5 (interferon regulatory factor 5), a transcription factor activated by TLR7, may play a major role in repressing ERV expression in vivo, given that mice defective for TLR7 spontaneously express ERVs [28].

More importantly, the authors found a spontaneous development of T-cell acute lymphoblastic leukemia (T-ALL) in aged mice triple-deficient for TLR3, TLR7 and TLR9. All T-ALL tumors showed a complex pattern of provirus integration, with signs of de novo insertion of activated ERV sequences [28]. TLR7-deficient mice showed an impaired adaptive immune response with a lack of ERV-specific IgG production. Interestingly, DCs from mice triple-deficient for TLR3, TLR7 and TLR9 were not able to prime natural killer (NK) cells to lyse a susceptible YAC-1 lymphoma cell line, suggesting impaired antitumor capacity in vitro. Overall, the authors show that TLRs play a crucial role in anti-ERV defense and that their loss results in T-ALL development in mice [28].

Furthermore, a very recent study showed the enhanced antitumor activity of a novel combination therapy (“shock and kill” strategy) based on HDACis and TLR7/8 agonists in human ovarian cancer cells. The HDACis were used to boost HERV expression in ovarian cancer cells (“shock”), and the TLR7/8 agonists provided the signal for apoptosis of susceptible HERV de-repressed tumor cells (“kill”), inducing a synergistic cytotoxic effect [38].

Double-stranded RNA from HERVs may also trigger the viral defense pathway through the activation of specific members of the RLR family, including RIG-I and melanoma differentiation-associated protein 5 (MDA5) [14]. RIG-I and MDA5 receptors are expressed in the cytoplasm of cells and their activation culminates with MAVS (mitochondrial antiviral signaling protein) interaction, and release of type I interferon, as well as expression of ISGs, which ultimately cause an immune activation status (Figure 1) [30,31,33].

In this scenario, several groups have focused their attention on the role of the induction of a viral mimicry state by increased transcription of HERV DNA in the setting of epigenetic therapy of cancer.

A recent study by Roulois et al. [29] showed that low-dose 5-aza-CdR increased the expression of selected HERVs in colorectal cancer cells, reducing the frequency of cancer-initiating cells (CICs) in primary colorectal cancer (CRC) both in vitro and in vivo, via activation of MAVS and IRF7.

In addition, a study by Chiappinelli et al. [39] showed that HERV upregulation occurred in epithelial ovarian cancer (EOC) cell lines following 5-azacytidine (5-aza-CR) and 5-aza-CdR treatment at day 7. Such upregulation did not lead to HERV protein expression but coincided with expression of ISGs, including IFNβ1, IRF7 and STAT1. In particular, a significant correlation between HERV transcripts and viral defense genes was observed in 19 primary ovarian tumors, with “high HERV” tumors significantly characterized by a high level expression of viral defense genes. Such an observation was further confirmed in primary EOC samples from The Cancer Genome Atlas (TCGA), showing a strong correlation with improved clinical prognosis [39].

Finally, a recent study confirmed that dual inhibition of DNA and histone methyltransferases induces a viral mimicry state, as well as cell death in ovarian cancer cell lines. In particular, G9a and DNA methylation were shown to repress a distinct set of HERVs and their combinatorial inhibition with G9ai plus 5-aza-CdR was able to activate more HERVs than the single-agent treatment [22].

However, high levels of HERV expression are also present in tumors characterized by constitutive DNA hypomethylation, such as testicular germ cell tumors (TGCTs). In particular, seminomas show variable DNA hypomethylation (i.e., 0.08% methylated CpG) or virtually complete demethylation. Moreover, a DNA hydroxymethylation can be found associated with the overexpression of TET (ten-eleven translocation) dioxygenases [40,41,42]. The pronounced DNA hypomethylation status observed in seminomas is correlated with a significant increase in HERV expression and IFN production, as well as CD8^+^ T cell infiltration [43].

Overall, the above mentioned studies support the notion that a marked DNA hypomethylation, that can be drug-induced or tumor-constitutive, may upregulate the expression of nucleic acid sequences (e.g., dsRNA) from HERV genomic regions, triggering a viral-like immune activation status that may potentiate the antitumor immune response.

## 4. HERV Antigens

The inherent dysregulation of the epigenetic state of the cancer genome may result in the expression of tumor-specific HERV proteins. These may provide a valuable pool of tumor-specific antigens (TSAs) able to elicit a potent adaptive immune response [44]. Indeed, HERV-derived antigens may activate both B cell and T cell responses in cancer patients (Figure 1). Therefore, the use of HERV TSAs in adoptive cell therapy, as well as in therapeutic cancer vaccines, is gaining significant interest [45,46].

From this perspective, a study by Wang-Johanning et al. [47] analyzed the immune response against HERV-K antigen in a cohort of breast cancer (BC) patients. HERV-K env protein was expressed in > 85% of human BC, with little or no expression in adjacent normal tissues or normal breast ductal tissues. High levels of IgG specific for HERV-K env protein were observed in approximately 50% of BC patients. Furthermore, an HERV-K-specific T cell response was observed in PBMCs from BC patients after in vitro stimulation with HERV-K env antigen, and a cytolytic activity against HERV-K targets could be detected.

A similar study showed expression of HERV transcripts and HERV-K env protein in ovarian cancer patients. High levels of IgG specific for HERV-K env protein were detected in sera, and T cells specific for HERV-K env epitopes were detected in PBMCs from patients with ovarian cancer [48].

Moreover, a recent study by Saini et al. [49] evaluated the presence of CD8^+^ T cell populations reactive to HERV-predicted peptides in PBMCs or BMMCs (bone marrow mononuclear cells) from 34 patients with hematological malignancies, including myelodysplastic syndrome (MDS), chronic myelomonocytic leukemia (CMML) and acute myeloid leukemia (AML), before and after 5-aza-CR treatment. The authors observed a significant enrichment of HERV-reactive T cells in 17 out of 34 patients. The HERV-specific T cell response strongly correlated with the expression of HERVs that were upregulated in patients independent of the 5-aza-CR treatment.

Finally, a study by Krishnamurthy et al. [50] evaluated the expression of HERV-K env protein in 220 melanoma samples from patients at various stages of disease. Immunohistochemical (IHC) analysis identified punctate cell surface expression and diffuse cytoplasmic staining on primary melanoma, supporting the notion that HERV-K env may represent a valuable target for adoptive cell therapy using engineered chimeric antigen receptor (CAR) T cells. Consequently, HERV-K env specific CAR T cells were generated and intravenously infused in a mouse model of metastatic HERV-K env^+^ melanoma. A significant reduction in tumor burden was observed 25 days after tumor injection, suggesting that engineered CAR T cells targeting an HERV-K env epitope may represent, indeed, a relevant strategy for the treatment of HERV-K env^+^ tumors [50].

Overall, these studies strongly support the notion that the expression of HERV antigens in cancer cells may induce high affinity B cell and T cell responses endowed with an antitumor effect, which is currently being evaluated in a phase I clinical trial (ClinicalTrials.gov Identifier: NCT03354390).

## 5. HERVs and Immune Checkpoint Blockade

Additional effects of HERV reactivation in tumor tissues are represented by T-cell infiltration, as well as upregulation of immune checkpoints, including programmed cell death-1 (PD-1) and cytotoxic T-cell-associated protein 4 (CTLA-4) [25]. Upregulation of CTLA-4 and PD-1 upon treatment with DNMT inhibitors (DNMTi) leads to increased sensitivity towards immune checkpoint inhibitors [39].

In line with this evidence, a seminal paper by Panda et al. [51] described the association between the expression of RNA transcripts from endogenous retroviruses and the response to immune checkpoint blockade (ICB) in renal cell carcinoma.

The correlation between the expression levels of 66 HERVs and immune checkpoint activation (ICA) was assessed in a cohort of 21 solid cancers from TCGA.

In particular, ICA was estimated by overall immune infiltration status and expression of CD8A, as well as the expression of genes of the pathways of PD-1, CTLA-4 and BTLA/HVEM (B and T lymphocyte attenuator/herpes virus entry mediator) [51].

A significant correlation between immune checkpoint activation and expression of HERVs was observed in clear cell renal cell carcinoma (ccRCC), breast cancer, colon cancer and head and neck squamous cell cancer (HNSC). However, the most significant association was observed for ccRCC, where the expression of 20 HERVs significantly correlated with ICA [51].

Interestingly, HERV expression levels defined three ccRCC subgroups (i.e., HERV-high, HERV-intermediate and HERV-low ccRCC) characterized by different levels of immune checkpoint activation. The HERV-high ccRCC subgroup showed high immune infiltration, including CD8^+^ T cells, follicular helper T cells, M1 macrophages, activated NK cells and plasma cells. On the contrary, M2 macrophages were more abundant in the HERV-low ccRCC subgroup [51].

Moreover, HERV-high ccRCCs showed a significantly higher expression of immune checkpoint genes (e.g., PD-1, PD-L1 (programmed death-ligand 1) and CTLA-4) compared with HERV-low ccRCCs [51].

Furthermore, the expression of ERV3-2 as a predictor of response to ICB was assessed in a validation cohort of 24 ccRCC patients. ERV3-2 RNA levels were significantly higher in tumors from responders compared with tumors from non-responders, supporting the notion that HERV expression may represent a good candidate predictor of response to ICB [51].

Finally, several epigenetic genes were identified whose expression levels were significantly correlated with overall HERV expression in ccRCC, breast cancer and colon cancer, strongly supporting the notion that epigenetic dysregulation may induce HERV reactivation in multiple cancers [51].

In such a complex scenario, several factors may contribute to an effective antitumor immune response, including HERV reactivation by epigenetic drugs. Indeed, HERV expression in tumor cells may activate a viral defense pathway similar to that observed upon viral infection. By activating HERVs, epigenetic drugs may turn “cold” tumors into “hot” tumors, promoting a robust immune cell infiltration. However, lymphocyte infiltration comes along with the upregulation of inhibitory immune checkpoint molecules that may, ultimately, cause the tumor to be more susceptible to immune checkpoint blockade [52].

Taken together, these results indicate that HERV reactivation significantly correlates with improved overall response in cancer patients treated with immune checkpoint inhibitors. This is currently being evaluated in several phase I/II clinical trials based on combination therapies, including epigenetic drugs and immune checkpoint inhibitors (ClinicalTrials.gov Identifier: NCT03220477, NCT03445858, NCT03903458, NCT04407741) [52].

## 6. HERVs and Liver Cancer

Hepatocellular carcinoma (HCC) is the most common primary liver malignancy. The major risk factors for HCC are hepatitis B virus (HBV) and hepatitis C virus (HCV) chronic infections, as well as chronic alcohol consumption [53]. Indeed, HCC development is a multistep process associated with genetic alterations, as well as dysregulated gene expression. However, recent findings indicate a dysregulated expression of LTR-derived noncoding RNAs (ncRNAs) in HCC [27,34].

In particular, the study by Hashimoto et al. [27] described a dysregulated expression of LTR-derived ncRNAs located at distal sites from protein-coding genes in tumor tissues from HCC patients with different etiologies (i.e., HBV, HCV, alcohol). The authors identified 20% activated LTR retroviral promoters in HCC. Interestingly, LTR activation was also observed in a mouse model of HCC.

Three classes of HCCs were defined according to LTR ncRNA expression levels (i.e., low, intermediate and high). In particular, LTR-high HCCs were significantly correlated with viral etiology (mostly HBV infection), high risk of recurrence and MYC pathway activation. On the contrary, LTR-low HCCs were mostly well differentiated, with lower risk of recurrence.

Similarly, a recent study by Wu et al. [34] described a novel lncRNA, namely, lncMER52A, a liver cancer-specific oncogenic lncRNA transcribed by MER52A LTR retrotransposon of the ERV1 class. The authors analyzed RNA-Seq datasets from 10 paired HCC tumor tissues and matched non-tumor tissues. Interestingly, lncMER52A was only expressed in HCC and not in its non-tumor counterpart and normal liver or normal tissues, except for testis and placenta. Interestingly, the authors found that increased levels of lncMER52A correlated with an advanced TNM stage, less differentiated tumors and shorter overall survival in HCC patients. LncMER52A was able to promote the invasion and metastasis of HCC cells in vitro and in vivo, regulating the EMT signaling pathway via post-translational control of p120-catenin protein stability.

Additionally, a specific HERV-K (HML-2) transcriptional activity was correlated with hepatoblastoma (HB) in children, as well as HCC in adults [54,55].

The association between the expression of RNA transcripts from HERV-K (HML-2) and pediatric liver malignancy was shown by RNA-Seq analysis [54]. The HERV-K RNA transcript profile was significantly variable across individual samples, with multiple proviruses transcribed from different loci in tumors from different patients. Moreover, the total number of expressed HERV-K proviruses at different loci was larger in HB samples than in controls. All expressed proviral loci were upregulated in HB compared to normal liver control (NC) samples, and five proviruses (i.e., 1q21.3, 3q27.2, 7q22.2, 12q24.33 and 17p13.1) were significantly differentially expressed (*p*-value < 0.05, log_2_ fold change > 1.5). HB samples were stratified according to HERV-K expression (i.e., high HERV-K HB vs. low HERV-K HB) and a differential gene expression analysis was performed. Overall, 775 differentially expressed genes were identified. Gene Ontology (GO) and Kyoto Encyclopedia of Genes and Genomes (KEGG) analyses indicated that cellular processes involved in leukocyte activation and immune responses were significantly enriched in HB samples with high HERV-K expression patterns [54].

Taken together, these results represent the first evidence of HERV-K (HML-2) expression in hepatoblastoma, and pave the way to further analyses of HERVs and pediatric tumors [54].

Similarly, the correlation between HERV-K (HML-2) expression and HCC was described in a recent paper by Ma et al. [55].

The expression of HERV-K (HML-2) RNA in HCC samples and adjacent non-tumor tissues was analyzed by qRT-PCR. HERV-K (HML-2) was significantly upregulated in HCC samples compared to adjacent non-tumor tissues (*p*-value < 0.01). Moreover, HERV-K (HML-2) expression in HCC was significantly associated with clinical parameters, including: cirrhosis, tumor differentiation and TNM stage (*p*-value < 0.05). 

HCC patients were stratified according to the normalized median level of HERV-K expression into low group (*n* = 42) vs. high group (*n* = 42) and a Kaplan–Meier analysis was performed. Interestingly, the group of patients with high-level expression of HERV-K showed poor prognosis with reduced overall survival (*p*-value < 0.01). The overall results support the notion that the upregulation of HERV-K in HCC may promote a malignant phenotype and a worse prognostic phenotype. However, the role of upregulated HERV-K (HML-2) in HCC and its correlation with underlying cell factors and/or signaling pathways is still poorly understood.

Although still in its infancy, these studies indicate that research on HERVs and HCC is gaining momentum. In particular, the inherent dysregulation of lncMER52A and HERV-K (HML-2) RNA observed in HCC may suggest their use as potential biomarkers or therapeutic targets.

## 7. Concluding Remarks

Current research on HERVs is gaining significant interest due to two major synergistically effects that HERV reactivation may have on the tumor-specific immune response in the setting of cancer therapy.

In particular, the viral mimicry state induced by HERV dsRNA may impact on the tumor-suppressive microenvironment, leading to an immune activation status with consequent tumor infiltration by immune cells. A second effect of HERV reactivation is associated with the expression of HERV antigens, which may ultimately evoke a vigorous tumor-specific B and T cell response.

Although still poorly understood, the potential role of HERVs in tumor growth and progression may guide the research and development of novel therapeutic approaches for cancer. From this perspective, additional studies are needed to fully characterize the effect of HERV reactivation on combinatorial strategies including epigenetic drugs and immune checkpoint inhibitors. In particular, the consistent expression of lncMER52A and HERV-K (HML-2) RNA in HCC lesions provides a strong rationale for the development of novel HCC-specific biomarkers, as well as therapeutic targets based on HERVs.

## Figures and Tables

**Table 1:** Classification of human endogenous retroviruses (HERVs). HERVs are grouped into three classes, based on similarity to the exogenous *Gammaretrovirus, Betaretrovirus* and *Spumavirus,* respectively.

Class	Family	Subgroups
Class I	HERV-H, HERV-F, HERV-W, HERV-R, HERV-P, HERV-E, HERV-I, HERV-T, ERV-FTD, ERV-FRD	
Class II	HERV-K	HML 1-11
Class III	HERV-L	

**Table 2:** Studies reporting HERV expression in cancer.

Cancer	HERV	Study Setting	Study Main Findings	Reference
Colorectal cancer	HERV	Preclinical	DNA-demethylating agents act by inducing endogenous dsRNAs that activate an interferon response pathway. This anti-viral response reduces proliferation of colorectal cancer-initiating cells.	[26]
Ovarian cancer	HERV families	Preclinical/Clinical	DNA methyltransferase inhibitors upregulate endogenous retroviruses in tumor cells to induce a growth-inhibiting immune response.	[27]
Ovarian cancer	HERV families	Preclinical	Dual inhibition of DNA and histone methyltransferases in ovarian cancer cell lines induces synergistic anti-tumor effects by upregulation of endogenous retroviruses, and activation of the viral defense response.	[15]
Breast cancer	HERV-K	Clinincal	HERV-K env protein products are able of acting as tumor associated antigens, activating both T cell and B cell responses in breast cancer patients.	[28]
Ovarian cancer	HERV-K	Clinincal	Ovarian cancer cells in primary tumors express HERV transcripts, including HERV-K env protein. Ovarian cancer patient sera contain HERV-K immunoreactive antibodies.	[29]
Melanoma	HERV-K	Preclinical/Clinical	HERV-K env protein is expressed on melanoma but not in normal tissues.	[30]
Renal cell carcinoma	HERV families	Clinincal	Abnormal expression of ERVs is associated with ccRCC, and ERV3-2 expression is associated with response to ICB in ccRCC.	[31]
Hepatoblastoma	HERV-K (HML-2)	Clinical	HERV-K is expressed from multiple loci in hepatoblastoma. Expression is increased for several proviruses compared to normal liver controls.	[32]
Hepatocellular carcinoma	HERV-K (HML-2)	Clinical	Upregulation of HERV-K (HML-2) in HCC patients is significantly correlated to cancer progression and poor outcome.	[33]

## Data Availability

The data presented in this study are available in the articles included in the reference list.
